# Sex Differences in Biopsy-Confirmed Diabetic Kidney Disease

**DOI:** 10.3389/fendo.2021.670674

**Published:** 2021-07-29

**Authors:** Yiting Wang, Jue Zhang, Junlin Zhang, Yucheng Wu, Rui Zhang, Honghong Ren, Mark E. Cooper, Fang Liu

**Affiliations:** ^1^Division of Nephrology, West China Hospital of Sichuan University, Chengdu, China; ^2^Department of Diabetes, Central Clinical School, Monash University, Melbourne, VIC, Australia

**Keywords:** sex differences, diabetic kidney disease, end stage kidney disease, risk factors, type 2 diabetes

## Abstract

**Background:**

To investigate the association between sex differences and end-stage kidney disease (ESKD) in patients with biopsy-confirmed diabetic kidney disease (DKD).

**Method:**

We performed a retrospective cohort study. A total of 336 patients with biopsy-confirmed DKD who were followed up for at least 12 months were enrolled. Baseline clinical and pathological data at the time of biopsy were collected. ESKD was defined by an estimated glomerular filtration rate of <15 ml/min/1.73 m^2^ or initiation of renal replacement therapy. The association between sex differences and ESKD was assessed using the log-rank test and Cox regression.

**Result:**

There were 239 (71%) male and 97 (29%) female patients in our cohort. Female patients had higher systolic blood pressure, total cholesterol and low-density lipoprotein cholesterol levels compared with male. There were a lower proportion of female patients in the very high risk grade according to the chronic kidney disease categories (37% of female vs. 44% of male). During a median follow-up time of 20 months, 101 (57.7%) male and 43 (44.3%) female entered into ESKD, with no significant difference by the log-rank test (*P >*0.05). Univariate [male: hazard ratio (HR) [95% confidence interval (CI)], 1.005, (0.702–1.439)] and multivariable ([male: HR (95%CI), 1.164, (0.675–2.007)]. Cox regression further showed that sex difference was not significantly associated with ESKD.

**Conclusion:**

Female patients had the higher systolic blood pressure, total cholesterol, LDL-C, compared with male patients. However, there was no significant association observed between sex difference and ESKD in our study.

## Introduction

Diabetic kidney disease (DKD) is one of the most common microvascular complications of diabetes. Despite improvements of management in basic research and clinical practice, DKD remains the leading cause of end-stage kidney disease (ESKD) worldwide (1, 2). In order to slow down the progression of DKD, recognizing patients with a high risk at an early stage is important. Sex differences have been taken into account in development or progression in several diseases such as diabetes (3), chronic kidney disease (CKD) (4), heart failure (5), and neuropsychiatric disorders (6). Recently, a study from the Chronic Renal Insufficiency Cohort that included 3,939 adults (half of them had diabetes) showed that male patients had the higher risk of CKD progression and death compared with female patients (4). Similarly, another large meta-analysis reported that males with CKD showed a more rapid decline in renal function than which in females, however, only patients with nondiabetic CKD were analyzed in that study (7).

The association between sex differences and the incidence or progression of DKD has been investigated in several studies, but not been well established with disparate conclusions (8). Different ethnic cohorts, age, type of diabetes and study designs can all cause the contradictory results. Moreover, most of the patients did not receive a kidney biopsy in these previous studies. Differences between DKD and nondiabetic kidney diseases greatly contribute to the challenges of understanding diabetic complications. Patients with nondiabetic kidney diseases might have confounded the results in previous study.

Therefore, in the current study, we aimed to investigate sex differences of clinical and pathological characteristics in patients with biopsy-confirmed DKD. We also aimed to evaluate the association between sex difference and ESKD.

## Method

### Study Design and Patients

We performed a retrospective cohort study. The study was approved by the ethics committee of West China Hospital of Sichuan University and all patients have signed a written informed consent form.

Patients with biopsy-confirmed DKD from January 2010 to December 2018 in our hospital were reviewed. Baseline data were collected from the hospital information system at the time patients received a kidney biopsy. The inclusion criteria were as follows: a. type 2 diabetes; b. biopsy-confirmed DKD; and c. follow-up for longer than 12 months (patients who developed ESKD in 12 months were also included). Type 2 diabetes was diagnosed in accordance with the 2018 American Diabetes Association criteria (9). Renal pathological classifications were based on the Renal Pathology Society in 2010 (10) by at least two professional pathologists. ESKD was defined as initiation of renal replacement therapy or eGFR less than 15 ml/min/1.73 m^2^.

### Statistical Analysis

Continuous variables were described as mean ± standard deviation (SD) or median and quartiles on the basis of a normality test. Categorical variables were presented as counts with ratios. Differences of baseline data between male and female patients were evaluated appropriately by the Student’s t test or the Mann–Whitney test. The prognosis of the kidney was compared by the log-rank test and shown using the Kaplan–Meier curve. Univariate and multivariate Cox analysis were applied to determine the risk factors of ESKD. All analyses were conducted using SPSS software 22.0 and GraphPad Prism 7.0. A two-sided *P*-value of less than 0.05 was considered statistically significant.

## Results

### Baseline Clinical and Pathological Characteristics

A total of 336 patients were enrolled in the study. Baseline clinical and pathological characteristics are shown in **Table 1**. Briefly, the mean age of patients was 51.7 ± 8.95 years old, 291 (86.6%) patients had hypertension, the median diabetic duration was 96 (36–141) months. The median eGFR was 59 (43–93) ml/min/1.73 m^2^ and the median proteinuria was 4.3 (2.0–7.8) g/24 h. There were 239 (71.1%) male and 97 (28.9%) female in our cohort; compared to male, female had the higher level of systolic blood pressure and lipid metabolism. Moreover, a significantly higher proportion of female patients received renin–angiotensin–aldosterone system (RAAS) inhibitors therapy. Male had the higher level of serum creatinine compared with female. There were no significantly differences in age, diastolic blood pressure, the duration of diabetes, the incidence of diabetic retinopathy, blood glucose, proteinuria, triglyceride, medical insurance, insulin use, statins and fibrates use. With regard to pathological lesions, 16 patients had glomerular class I, 77 had class IIa, 33 had class IIb, 153 had class III, and 52 had class IV. However, there were no significant differences in glomerular class, interstitial fibrosis and tubular atrophy (IFTA), interstitial inflammation and arteriolar hyalinosis between male and female patients.

**Table 1:** Baseline clinicopathological findings in male and female groups.

Variables	Total (n = 336)	Male (n = 239)	Female (n = 97)	*P* value
**Age (years)**	51.7 ± 8.95	51.5 ± 8.92	52.1 ± 9.06	>0.05
**Body mass index (kg/m^2^)**	25.7 (23.2–27.7)	25.7 (23.2–27.8)	25.4 (23.2–27.5)	>0.05
**Current Smoker (n, %)**	107 (32)	103 (43)	4 (4)	<0.001
**Hypertension (n, %)**	291 (86.6)	202 (84.5)	89 (91.8)	>0.05
**Systolic blood pressure (mmHg)**	145 ± 23.1	143 ± 22.5	152 ± 23.4	0.001
**Diastolic blood pressure (mmHg)**	86 ± 13.2	85 ± 12.1	88 ± 15.7	>0.05
**Diabetes duration (months)**	96 (36–141)	96 (36–144)	96 (36–132)	>0.05
**Diabetic retinopathy (n, %)**	153 (47) (n = 327)	105 (46) (n = 230)	48 (49) (n = 97)	>0.05
**Fasting blood glucose (mmol/L)**	8.3 ± 4.16	8.2 ± 3.99	8.6 ± 4.54	>0.05
**Glycosylated hemoglobin (%)**	7.3 (6.2–8.6)	7.4 (6.3–8.6)	7.2 (6.1–8.4)	>0.05
**Serum albumin (g/L)**	34.3 ± 7.74	34.7 ± 7.57	33.2 ± 8.08	>0.05
**Hemoglobin (g/L)**	120.2 ± 27.9	125.4 ± 28.7	107.1 ± 20.7	<0.05
**Serum creatinine (umol/L)**	119 (80–158)	127 (89–163)	96 (70–137)	<0.001
**eGFR (ml/min/1.73 m^2^)**	59 (43–93)	58 (43–92)	61 (42–94)	>0.05
**BUN (mmol/L)**	9.1 ± 5.42	9.1 ± 4.01	9.1 ± 7.92	>0.05
**Proteinuria (g/24 h)**	4.3 (2.0–7.8) (n = 306)	4.3 (2.2–7.8) (n = 214)	4.3 (1.8–7.7) (n = 92)	>0.05
**Triglyceride (mmol/L)**	2.20 ± 1.757	2.09 ± 1.562	2.45 ± 2.152	>0.05
**Total cholesterol (mmol/L)**	5.24 ± 1.63	4.96 ± 1.45	5.92 ± 1.84	<0.001
**LDL-C (mmol/L)**	3.08 ± 1.267	2.93 ± 1.144	3.46 ± 1.470	0.002
**HDL-C (mmol/L)**	1.34 ± 0.541	1.28 ± 0.532	1.49 ± 0.534	0.001
**Uric acid (mmol/L)**	383 (337–434)	397 (349–451)	354 (311–391)	<0.001
**Medical insurance (n, %)**	223 (66.4)	158 (66.1)	65 (67.0)	>0.05
**Pathological lesions (n = 331)**				
**Glomerular class (n = 331)**				>0.05
**I**	16	12	4	
**IIa**	77	57	20	
**IIb**	33	25	8	
**III**	153	04	49	
**IV**	52	36	16	
**Interstitial fibrosis and tubular atrophy**				>0.05
**0**	10	8	2	
**1**	141	93	48	
**2**	139	103	36	
**3**	41	31	10	
**Interstitial inflammation**			n = 96	>0.05
**0**	20	17	3	
**1**	241	161	80	
**2**	70	57	13	
**Arteriolar hyalinosis**				>0.05
**0**	32	23	9	
**1**	170	125	45	
**2**	129	87	42	
**Use of medications**				
**RAAS inhibitors (n, %)**	267 (79.5)	183 (76.6)	84 (86.6)	0.039
**Insulin use (n, %)**	240 (71.9)	166 (69.7)	74 (77.1)	>0.05
**Statins (n, %)**	193 (57.4)	129 (54.0)	64 (66.0)	>0.05
**Fibrates (n, %)**	15 (4.5)	10 (4.2)	5 (5.2)	>0.05
**Follow-up information**				
**Follow-up duration (months)**	20 (14–35)	19 (13–35)	23 (14–36)	>0.05
**ESKD (n, %)**	144 (57.1)	101 (57.7)	43 (44.3)	>0.05

Data are presented as the mean ± standard or counts and percentages.

eGFR, estimated glomerular filtration rate; BUN, blood urea nitrogen; HDL-C, high density lipoprotein cholesterol; LDL-C, low-density lipoprotein cholesterol; RAAS, Renin–angiotensin–aldosterone System; ESKD, end stage kidney disease.

A two-tailed p <0.05 was considered statistically significant.

### Metabolic Characteristics Between Male and Female Patients

With regard to metabolic characteristics, the body mass index (male vs. female 25.7 (23.2–27.8) kg/m^2^ vs. 25.4 (23.2–27.5) kg/m^2^, *P >*0.05) and triglyceride (male vs. female 2.09 ± 1.562 mmol/L vs. 2.45 ± 2.152 mmol/L, *P >*0.05) were not significant different between male and female. However, compared with male patients, female patients had the significantly higher total cholesterol (male vs. female 4.96 ± 1.45 mmol/L vs. 5.92 ± 1.84 mmol/L, *P <*0.05), LDL-C (male vs. female 2.93 ± 1.144 mmol/L vs. 3.46 ± 1.470 mmol/L, *P <*0.05), HDL-C (male vs. female 1.28 ± 0.532 mmol/L vs. 1.49 ± 0.534 mmol/L, *P <*0.05), but lower uric acid (male vs. female 397 (349–451) mmol/L vs. 354 (311–391) mmol/L, *P <*0.05).

### CKD Risk Categories

**Figure 1:**
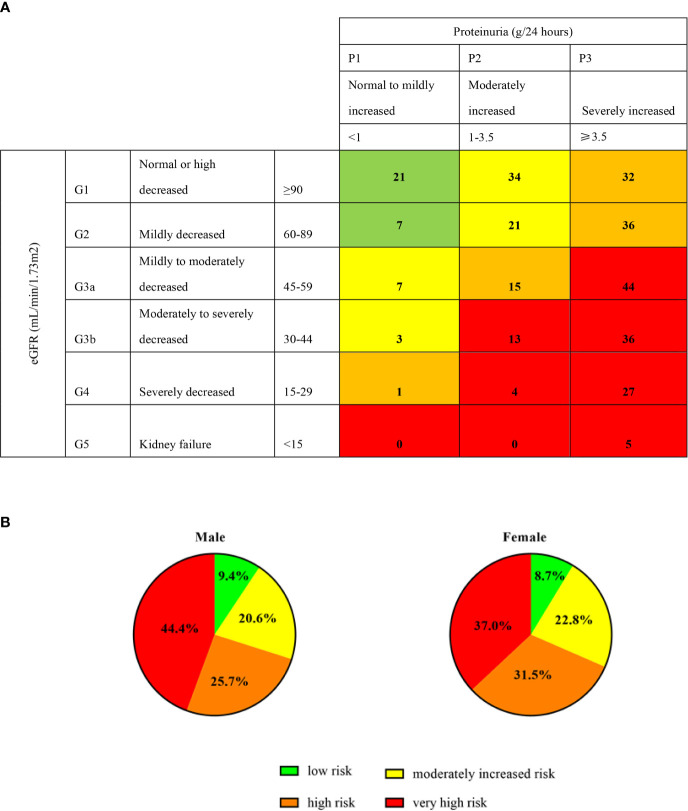
Prognosis of CKD categories and sex. Proteinuria (g/24 hours) of 306 patients were obtained at the baseline. Green, low risk (if no other markers of kidney disease, no CKD); Yellow: moderately increased risk; Orange: high risk; Red, very high risk. The digits in **(A)** cells represent the numbers of patients. **(B)** represent the percentage of male and female in different categories.

To evaluate the risk distribution between male and female patients, we used the CKD category heat map as recommended by the Kidney Disease Improving Global Outcomes (11). Patients were categorized into low risk (green), moderately increased risk (yellow), high risk (orange) and very high risk (red) grades by baseline proteinuria (24 hour-proteinuria of 306 patients were obtained) and eGFR. Those patients in the red category had the highest proteinuria and lowest GFR, and carried highest risk for events of cardiovascular disease, ESKD and mortality. A total of 9% (28/306) of patients were low risk, 21% (65/306) of patients had a moderately increased risk, 27% (84/306) were high risk, and 42% (129/306) were very high risk (**Figure 1A**). As for sex distribution, both approximately 30% of male and female patients had low and moderately increased risks, but more male had a higher risk than female (44% vs. 37%) (**Figure 1B**).

### Sex Difference and ESKD

**Figure 2:**
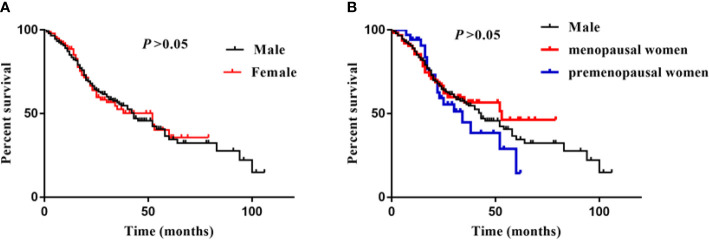
Sex difference and ESKD. **(A)** was showed survival curves of male and female, **(B)** was showed survival curves of male, menopausal/premenopausal women. Log-rank analysis was used to compared the percent survival between male and female. There was no significant difference between male, premenopusal and menopausal female.

During a median follow-up period of 20 (14–35) months, a total of 144 (57.1%) patients developed ESKD. Specifically, there were 101 (57.7%) male, 18 (52.9%) premenopausal female, and 25 (39.7%) menopausal female suffered from ESKD during the follow-up time. There was no significant difference in kidney survival between male and female, and no difference between premenopausal and menopausal female (**Figure 2**).

To evaluate risk factors of ESKD in patients with DKD, we performed univariate and multivariable Cox regression analyses (**Table 2**). Specifically, the hazard ratio (HR) and 95% confidence interval (CI) of male was 1.005 (0.702–1.439, *P* = 0.978), which indicated there was no association between sex and ESKD. The higher levels of systolic blood pressure, proteinuria, total cholesterol, LDL-C, HDL-C, advanced class of glomerular lesion, IFTA, interstitial inflammation, arteriolar hyalinosis, incidence of diabetic retinopathy, and the lower levels of serum albumin and eGFR were associated with ESKD. Moreover, when we adjusted for essential clinical and pathological indices, sex was still not associated with ESKD (HR and 95% CI, 1.164, 0.675–2.007, *P* = 0.584). However, a higher levels of interstitial inflammation (HR and 95% CI, 1.705, 1.041–2.791, *P* = 0.034), and the lower serum albumin (HR and 95% CI, 0.895, 0.858–0.932, *P <* 0.001) and eGFR (HR and 95% CI, 0.969, 0.959–0.979, *P <* 0.001) were independently associated with ESKD.

**Table 2:** Risk factors of ESKD.

Variables	HR	95% CI	*P* value	HR	95% CI	*P* value
	Univariate			Multivariate		
**Male**	1.005	0.702–1.439	0.978	1.164	0.675–2.007	0.584
**Age**	0.991	0.973–1.008	0.303	0.975	0.951–0.999	0.043
**Systolic blood pressure**	1.007	1.000–1.015	0.045	0.995	0.986–1.005	0.341
**Current smokers**	0.900	0.634–1.278	0.556	0.850	0.500–1.446	0.549
**Diabetes duration**	1.002	0.999–1.004	0.171	1.001	0.998–1.005	0.466
**HbA1c**	0.927	0.842–1.021	0.125	0.955	0.863–1.058	0.381
**Diabetic retinopathy**	1.876	1.344–2.619	<0.001	1.487	0.948–2.333	0.084
**Serum albumin**	0.899	0.879–0.920	<0.001	0.895	0.858–0.932	<0.001
**eGFR**	0.968	0.961–0.975	<0.001	0.969	0.959–0.979	<0.001
**Proteinuria**	1.104	1.076–1.134	<0.001	1.003	0.944–1.066	0.919
**Triglyceride**	0.935	0.842–1.039	0.211	0.817	0.598–1.116	0.205
**Total cholesterol**	1.184	1.075–1.303	0.001	1.606	0.777–3.320	0.201
**LDL-C**	1.221	1.081–1.378	0.001	0.537	0.260–1.111	0.094
**HDL-C**	1.320	1.017–1.712	0.037	0.703	0.318–1.552	0.383
**Glomerular class**	1.758	1.482–2.085	<0.001	1.029	0.782–1.354	0.837
**IFTA**	1.813	1.456–2.257	<0.001	0.769	0.519–1.139	0.190
**Interstitial inflammation**	2.938	2.105–4.100	<0.001	1.705	1.041–2.791	0.034
**Arteriolar hyalinosis**	1.518	1.171–1.967	0.002	1.103	0.771–1.578	0.590

eGFR, estimated glomerular filtration rate; LDL-C, low-density lipoprotein cholesterol; HDL-C, high density lipoprotein cholesterol; IFTA, interstitial fibrosis and tubular atrophy; HR, Hazard ratio; CI, confidence interval. Univariate and multivariate indicated that sex was not associated with ESKD.

A two-tailed p < 0.05 was considered statistically significant.

## Discussion

DKD has become the leading cause of ESKD, which has led to a heavy economic burden on individuals and countries (2). Therefore, recognizing risk factors of ESKD would be beneficial for to slowing the progression of DKD. The association between sex difference and ESKD in patients with DKD has not been well established. In the current study, the proportion of male was higher than that of female. We also found that male patients had relatively good control of lipid metabolism. However, more male patients were in the high very risk grade of CKD categories at baseline compared with female. However, there was no association between sex difference and ESKD in our study.

Increasing studies have investigated the effect of sex differences on DKD development and progression, however, but different cohorts have reported conflicting findings. In studies that enrolled patients with type 2 diabetes, it seems that more results indicated female has greater risk of DKD progression (8). A study from Japan (12) (247 male and 97 female) showed that the mean annual decline in the eGFR was 3.5% in female and 2.0% per year in male. However, this study only enrolled patients with diabetes or those at the early stage of CKD (mean eGFR >90 ml/min/1.73 m^2^, only 28.5% of patients had proteinuria). Similarly, several studies showed that African American, Hispanic and Pima Indian female had a higher risk of DKD and disease progression (13–15). Nevertheless, another prospective observational study (227 male and 60 female) enrolled patients with type 2 diabetes and persistent macroalbuminuria (≥300 mg/24 h) and showed that sex difference had no association with DKD progression (16). This previous finding is similar to our results. In our study, the ratio of male and female (approximately 2.5) was consistent with previous studies, but patients with the lower eGFR and greater proteinuria. Moreover, studies have found that the effect of sex is less apparent in DKD than in non-DKD (17, 18). Our patients with DKD were all diagnosed by a kidney biopsy, which excluded non-DKD, and this could explain the results.

The recognition of underlying mechanism of sex differences in diseases remains limited. Sex hormones are considered to be the main driver of sex disparities in the incidence and progression of CKD. A meta-analysis that included 11,345 patients clearly indicated that male was associated with a faster progression of nondiabetic CKD (7). However, this renoprotective effect was only evident in premenopausal female (19, 20). Once patients suffer from diabetes, the renoprotective effect of female is generally considered lost, even in premenopausal female (21). Accumulating evidence suggests that patients with diabetes have unbalanced levels of sex hormones, where expression of estradiol is decreased, but testosterone is increased, in female with diabetes (22, 23). Moreover estrogen replacement alleviates pathological lesions in animal DKD models (24–26), and can even attenuate proteinuria and improve creatinine clearance in postmenopausal female with diabetes (27). In our study, most of female were during perimenopause which worsened the imbalance of hormones. This could also explain why there was no significantly difference in kidney survival among premenopausal, menopausal female and male in our cohort.

There are other possible mechanisms contribute to sex differences. Studies have suggested that more adolescent female with diabetes had hyperfiltration in the early stage of DKD (28, 29). Additionally, the higher baseline total cholesterol and LDL-C of our female patients, which was consistent with a cohort from Australia (30), also increased the risk of hyperfiltration. Hyperfiltration traditionally indicates a poor kidney prognosis, but a recent study from the Diabetes Control and Complications Trial/Epidemiology of Diabetes Interventions and Complications (DCCT/EDIC) followed up 446 patients with type 1 diabetes for longer than 20 years found that early hyperfiltration was not associated with decreased renal dysfunction (31). Therefore, although female patients with diabetes are more likely to have hyperfiltration, this does not affect kidney prognosis. The expression and mechanism of several therapeutic targets had been found different between male and female. Specifically, some studies have observed that male had the higher expression of ANG II (32, 33), and ANG II is recognized to mediate renal inflammation (34). Additionally, the expressions of sodium-glucose co-transporters (SGLT) 1 and SGLT2 have been found higher in female rats than in male rats (35, 36). A recent meta-analysis also showed that a reduction in major adverse cardiac events with SGLT2 inhibitors was less in female with diabetes compared with male with diabetes (37). The underlying mechanisms of these differences remain unclear, but it is worthy to be further investigated to provide individual therapy and improve prognosis of patients with diabetes.

There were several limitations should be addressed. First, this was a retrospective cohort study, and we only observed the relationship between sex differences and kidney prognosis. therefore, prospective studies are warranted to determine the underlying causative relationship. Second, our study only included Chinese patients, various genetic backgrounds might have affected our results. Third, we had no opportunity to evaluate the levels of sex hormones at baseline owing to the study design. Fourth, the sample size was limited and patients were in a relatively severe disease stage because we only enrolled patients with biopsy-confirmed DKD. Therefore, further prospective and large sample size DKD cohorts are required to investigate the issue.

## Conclusion

In patients with biopsy-confirmed DKD, female patients had the higher systolic blood pressure, total cholesterol, LDL-C levels, compared with male patients. However, there was no significant association was observed between sex difference and ESKD in our study.

## Perspectives And Significance

Sex differences play an important role in many diseases including cancers or chronic diseases. However, the association between sex differences and the incidence or progression of DKD has not been well established with disparate conclusions. Therefore, we investigate the issue in patients with biopsy-confirmed DKD. We found that female patients had the higher systolic blood pressure, total cholesterol, LDL-C levels. However, there was no association between sex difference and ESKD in our study. The study provides relatively strong evidence to illustrate the associations.

## Data Availability Statement

The raw data supporting the conclusions of this article will be made available by the authors, without undue reservation.

## Ethics Statement

The studies involving human participants were reviewed and approved by ethics committee of West China Hospital of Sichuan University. The patients/participants provided their written informed consent to participate in this study.

## Author Contributions

YTW, JZ, and FL planed, analyzed and wrote the manuscript. JLZ, YCW, RZ, and HR collected data, check analysis and gave some suggestions. MC revised the manuscript and gave lot of suggestions. All authors contributed to the article and approved the submitted version.

## Funding

This study was supported by Grant 8197031494 from the National Natural Science Foundation of China.

## Conflict of Interest

The authors declare that the research was conducted in the absence of any commercial or financial relationships that could be construed as a potential conflict of interest.

## Publisher’s Note

All claims expressed in this article are solely those of the authors and do not necessarily represent those of their affiliated organizations, or those of the publisher, the editors and the reviewers. Any product that may be evaluated in this article, or claim that may be made by its manufacturer, is not guaranteed or endorsed by the publisher.
